# Child maltreatment and quality of life: a study of adolescents in residential care

**DOI:** 10.1186/s12955-016-0479-6

**Published:** 2016-05-10

**Authors:** Hanne Klæboe Greger, Arne Kristian Myhre, Stian Lydersen, Thomas Jozefiak

**Affiliations:** Department of Child and Adolescent Psychiatry, St. Olavs Hospital, Pb 6810 Elgeseter, 7433 Trondheim, Norway; Department of Public Health and General Practice, Norwegian University of Science and Technology (NTNU), Faculty of Medicine, Pb 8905 MTFS, 7491 Trondheim, Norway; Children’s Clinic, St.Olavs Hospital, Pb 3250 Sluppen, 7006 Trondheim, Norway; Norwegian University of Science and Technology (NTNU), Faculty of Medicine, RKBU Central Norway, Pb 8905 MTFS, 7491 Trondheim, Norway

**Keywords:** Child abuse, Maltreatment, Adolescents, KINDL-R, Quality of life, Residential care

## Abstract

**Background:**

Childhood maltreatment is an important risk factor for mental and physical health problems. Adolescents living in residential youth care (RYC) have experienced a high rate of childhood maltreatment and are a high-risk group for psychiatric disorders. Quality of life (QoL) is a subjective, multidimensional concept that goes beyond medical diagnoses. There is a lack of research regarding the associations between childhood maltreatment and QoL. In the present study, we compare self-reported QoL between adolescents in RYC in Norway with and without maltreatment histories, and adolescents from the general population. We also study the impact of number of types of adversities on QoL.

**Methods:**

Adolescents aged 12–23 years living in RYC in Norway were invited to participate in the study; 400 participated, yielding a response rate of 67 %. Maltreatment histories were assessed through interviews with trained research assistants, and completed by 335 adolescents. Previous exposure to maltreatment was reported by 237 adolescents. The Questionnaire for Measuring Health-Related Quality of Life in Children and Adolescents (KINDL-R) was used. Nonexposed peers in RYC (*n* = 98) and a sample of adolescents from the general population (*n* = 1017) were used for comparison. General linear model analyses (ANCOVA) were conducted with five KINDL-R life domains as dependent variables. Linear regression was used to study the effect of number of types of adversities.

**Results:**

Exposed adolescents in RYC reported poorer QoL than peers in control groups. Compared with nonexposed peers in RYC, the 95 % confidence intervals for mean score differences on the KINDL-R subdomains (0–100 scale) were 1.9–11.4 (Physical Well-being), 2.2–11.1 (Emotional Well-being), −0.7–10.0 (Self-esteem), and 1.8–10.9 (Friends). Compared with the general population sample, the 95 % confidence intervals for mean score differences were 9.7–17.6 (Physical Well-being), 7.9–15.3 (Emotional Well-being), 3.6–12.5 (Self-esteem), and 5.3–12.8 (Friends). Number of types of adversities was associated with a poorer QoL score on all subdomains (Physical- and Emotional Well-being, Self-esteem, Friends, and School).

**Conclusions:**

Childhood maltreatment was associated with a poorer QoL score. We suggest the use of QoL and maltreatment measures for all children and adolescents in RYC.

## Background

Child maltreatment can have devastating effects for those exposed. International research has revealed an increased risk of reduced self-reported health and life satisfaction [1, 2] and increased prevalence of migraines [3], overweight [4], asthma [5], gastrointestinal illness [6], and psychosocial problems, including reduced school functioning, in adolescence and in early adulthood in individuals with child maltreatment histories, such as physical abuse, sexual abuse, and witnessing violence [7–9]. Adolescents and adults who have been exposed to maltreatment during childhood are also at increased risk of a broad spectrum of psychiatric illnesses, such as depression, anxiety, suicide ideation, eating disorders, conduct disorders, and drug abuse [10–14]. In addition, there is a growing body of literature showing that polyvictimization increases the risk of several psychiatric disorders and symptoms [14–16].

Reported prevalence rates of childhood maltreatment vary in different studies and in different countries. In the Norwegian general population, studies have reported a prevalence of physical abuse of 5–6 % (both sexes) and sexual abuse of 10–14 % (girls) and 3–4 % (boys) [17, 18]. In a meta-analysis of child sexual abuse prevalence in European countries from 2011, similar results are reported (14 % of girls and 6 % of boys) [19]. However, the Adverse Childhood Experiences (ACE) study in the United States found higher rates of maltreatment, with a prevalence of childhood physical abuse of 27 % (girls) and 30 % (boys), and a prevalence of child sexual abuse of 25 % (girls) and 16 % (boys) [20]. The childhood prevalence of witnessing intimate partner violence in high-income countries has been estimated as 8–25 % [21].

Adolescents in residential youth care (RYC) are at high risk of developing psychiatric disorders [22, 23]. In a recently published article, we found that the maltreated adolescents in RYC had a very high prevalence of psychiatric diagnoses (80 %) compared with the nonmaltreated adolescents (64 %) in RYC, and that exposure to increasing numbers of types of childhood adversities significantly increased the risk of having several psychiatric diagnoses according to the Diagnostic and Statistical Manual of Mental Disorders, 4^th^ Edition (DSM-IV) [24]. It has also been shown that mental health problems are associated with poorer quality of life (QoL) [23, 25], and that adolescents in RYC report poor QoL [26]. The World Health Organization (WHO) defines QoL as “individuals’ perception of their position in life in the context of the culture and value systems in which they live and in relation to their goals, expectations, standards and concerns” [27]. In children, this would include the child’s own experiences across several life domains, such as physical and emotional well-being, self-esteem, and the child’s relationship to family, friends, and school [28]. The WHO highlights that the concept of QoL is subjective and multidimensional. Therefore, self-reports are the gold standard of QoL assessment. However, in this study, we have chosen to include proxy reports of QoL as well, for additional information. Even though psychiatric and somatic symptoms and disorders are related to the individual’s QoL, the term does not address psychopathology directly, but rather the impact of psychopathology on subjective perceived daily function. It also includes other factors, such as self-esteem and the person’s relationship to family, friends, and school. Previous studies have shown that QoL and psychopathology are distinct features, and that it is possible to improve QoL measures even with persistent high levels of psychopathology [29]. Improvement in QoL or daily function provides an alternative outcome goal for clinicians working with children and adolescents with chronic psychiatric or somatic disorders. Adolescents in RYC with child maltreatment histories are at high risk of developing problems in several life domains, and it can be challenging to find the most beneficial treatment or prevention programs for each individual. The use of QoL measures could give important information that would go beyond the diagnosis of disease, and suggest which life domain could be targeted for interventions and improvement of functioning. As the caregivers of all children and adolescents in out-of-home care, child welfare services are responsible for providing safe environments and looking after the health of the children in their care. Considering existing research on maltreatment, one would therefore expect that child well-being should be of great interest to child welfare services. However, while there has been an increasing amount of research regarding the mental health of children in out-of-home care, research on the QoL of children in out-of-home care has been very sparse [26].

The previous research on the QoL of maltreated children and adolescents has also been very limited [30]. In a recently published review, Weber et al. concluded that there is a consistently negative association between child maltreatment and both self- and proxy-reported QoL. They also found that the number of types of maltreatment and QoL were negatively related, although the studies that had investigated this all assessed adult survivors rather than children [31]. A recent study of Swedish 15-year-old school children found a dose–response relationship between the number of types of abuse (not including sexual abuse) and a decrease in a QoL measure [32]. In a study of Chinese adolescents, Chan reported that youth exposed to polyvictimization reported poorer health-related QoL than nonvictimized peers [33]. In a large study of high school students in Kuwait, Al-Fayez et al. reported significantly poorer QoL in students exposed to maltreatment [34]. A few other studies have focused on the QoL of maltreated children and the QoL of adults who have experienced childhood maltreatment. Adult survivors of childhood maltreatment have shown significant loss of health-related QoL and of remaining quality-adjusted life years [35, 36]. Lanier et al. reported that children who received child welfare services as a follow-up to a report of child abuse or neglect had significantly lower QoL scores compared with a normative reference group [37]. A Swiss study of the health-related QoL of young maltreated children (mean age 8 years) also showed a significantly impaired QoL among maltreated children compared with matched controls [38].

The existing lacuna in research on QoL in RYC adolescents with maltreatment histories is of major concern because such knowledge could illuminate the life domains that could be targets for intervention and used to support the adolescent beyond the assessment and treatment of psychopathology. The primary aim of this study was to assess the QoL of adolescents in RYC units who have reported previous experience of maltreatment, and to compare them with adolescents in the same RYC units without this experience, and with adolescents from the general population. We also wanted to study the impact of the number of types of adversities on QoL scores for different subdomains. In addition to adolescent self-reports, proxy reports by primary contacts were assessed as a supplement.

## Method

### Participants and recruitment

#### The study sample

**Fig. 1:**
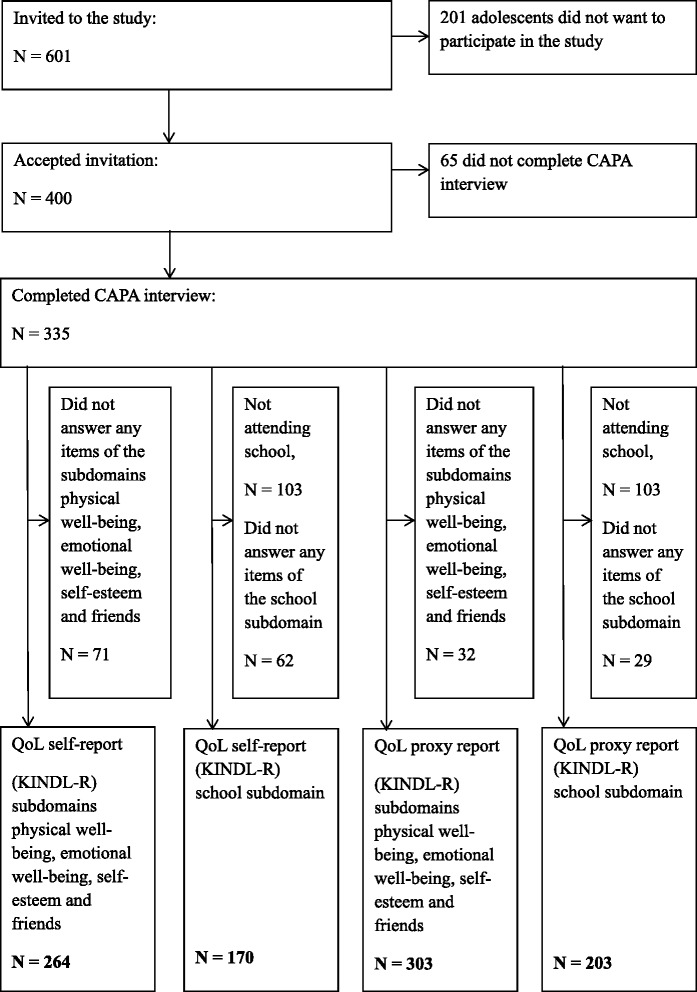
Flow-chart for inclusion in RYC sample

The data for this study were obtained from the Norwegian research project, Mental Health in Adolescent Residents in the Child Welfare System [22]. All residential care units providing care for adolescents aged 12–23 years in Norway were invited to participate in the study (Fig. 1). Unaccompanied minors without asylum in Norway and adolescents on acute placement were excluded from the study because they were considered to be in such a high state of crisis that data collection should not be prioritized. Insufficient Norwegian language ability was another exclusion criterion. A total of 86 of the 98 invited institutions agreed to participate in the study, and 400 of the 601 eligible adolescents participated, giving a response rate of 67 %. Of those included in the study, 335 youths completed the psychiatric interview, yielding information about child maltreatment histories. Child Behavior Check List (CBCL) scores were available for the participants, as well as for 141 anonymous nonparticipants. These data made it possible to estimate complete DSM-IV diagnoses for 541 adolescents using Bayesian multiple imputation estimation [22]. Estimated prevalence rates of psychiatric diagnoses showed only a small deviance from the observed prevalence rates, which were based on completed psychiatric interviews, thereby confirming the representativeness of the 335 youths who completed the psychiatric interview. For further details, see Greger et al. [24] and Jozefiak et al. [22].

#### The general population reference sample

As a comparison group, we used a study sample of students from 4th to 10th grade from schools in Sør-Trøndelag county, which is a geographical area representative of Norway in general, with both urban and rural settlement. In this study, 61 school grade cohorts in the chosen geographical area were randomly selected (a school grade cohort was defined as all pupils enrolled in a specific grade at a single school). Students with limited Norwegian language skills or with a low academic developmental level were excluded. Students in the 4, 6, 8 and 10th grades were invited to participate in the study, and 1997 students were finally included, resulting in a response rate of 71.2 %. For the present study, only data from students aged 12 years and older were used (Table 1). Students and their parents completed the KINDL-R (see below) independently. For further details, see Jozefiak et al. [39].

**Table 1:** Characteristics of the study sample and reference groups

	Adolescents in RYC reporting exposure to maltreatment	Adolescents in RYC reporting no exposure to maltreatment	General population
Number	237	98	1017
Age (years)			
Mean (SD)	17.0 (1.31)	16.5 (1.30)	14.1 (1.02)
Range	12–20	12–19	12–17
Sex			
Girls	64.6 %	43.9 %	49.5 %
Mean number of placements out of family home	3.6	2.9	
Mean age at first placement, years	12.8	12.0	
Witnessing violence	38.4 %	0 %	
Victim of physical violence, family	54.4 %	0 %	
Victim of physical violence, community	34.2 %	0 %	
Sexual abuse	37.6 %	0 %	
Household dysfunction	66.7 %	46.9 %	
>1 type of childhood adversity^a^	66.2 %	0 %	

*Note:* ^a^ Types of adversities defined by witnessing violence, victim of family violence, sexual abuse, household dysfunction

### Procedures

Data collection was conducted by research assistants who visited the institutions and completed structured psychiatric interviews with adolescents and their primary contacts, and collected questionnaires from adolescents, primary contacts, and leaders of the institutions. Four trained interviewers were used; they had been educated in relevant fields (Master’s degrees in psychology/social work, one Bachelor’s degree in mental health, and a nurse who specialized in mental health) and had extensive prior experience working with children and families. During the whole period of data collection, a team of child and adolescent psychiatrists and psychologists was on call in case of emergencies. Data were collected from June 2011 until July 2014.

### Instruments

#### Child and Adolescent Psychiatric Assessment (CAPA)

The CAPA is a semistructured psychiatric interview designed to gather information from children and adolescents [35]. The CAPA uses a computer-based algorithm for diagnostic evaluation, which results in DSM-IV diagnoses. It also contains questions about child abuse history. The following specific items from the CAPA were used to extract the data of interest for this study:

1. *Witness of violence* – Person saw or heard but was not the object of an event with potential for life threatening or severe physical injury, including seeing someone shot or killed, hearing someone raped or beaten in an adjacent room, or seeing another person be killed or severely injured in an accident.
2. *Victim of violence in the community* – Subject has been the victim of physical violence, with one or more people (not a family member) using force against them with potential to cause death or serious injury. Force may have been used in order to get something (e.g., mugging or robbery), or to intimidate or frighten the subject, or for its own sake (assault, fight, or torture). Victim may have been threatened with a weapon.
3. *Victim of family violence* – Subject has been the victim of physical abuse by a member of the family.
4. *Victim of sexual abuse* – Sexual abuse episode(s) in which a person (“the perpetrator”) involved the child or adolescent in activities for the purpose of the perpetrator’s own sexual gratification. Activities included kissing (that made the person uncomfortable), genital fondling (over or under clothing), oral–genital or oral–anal contact, genital or anal intercourse, or use of instruments. Sexual abuse does not include medical exams or mutually desired sexual relations with a peer.

All participants who indicated that they had experienced at least one of these types of maltreatment were included in the exposed group (*n* = 237). The participants that did not respond positively to these questions were used as a reference group of adolescents in residential care (*n* = 98).

#### Childhood adversity scale: measuring the impact of number of types of adversities

One of the aims of this study was to investigate the impact of number of types of adversities on different aspects of QoL. We constructed a modified scale based on the ACE questionnaire [40], where the number of maltreatment types we had registered, including household dysfunction, was added. The term “household dysfunction” will hereafter represent parental psychopathology, criminality, or alcohol or substance abuse. Confirmatory factor analysis was conducted, and after excluding the variable “victim of violence in the community,” our scale showed a one-factor structure with good model fit to the data: root mean square error of approximation (RMSEA) = 0.00 with a 90 % confidence interval (CI) of 0.00 to 0.08, comparative fit index (CFI) = 1.00, and Tucker–Lewis index (TLI) = 1.00. Thus, a scale (range 0–4) describing the load of childhood adversities was developed, and consisted of the variables of witnessing violence, victim of family violence, victim of sexual abuse, and household dysfunction. The procedure is described in more detail elsewhere [24].

#### Quality of life (QoL)

The Kinder Lebensqualität Fragebogen (Questionnaire for Measuring Health-related Quality of Life in Children and Adolescents, revised version, KINDL-R) is a well-established QoL instrument for children aged 8–16 years used in several clinical and epidemiological studies. A parent-proxy version is available. The questionnaire consists of 24 items and six subscales: Physical Well-being, Emotional Well-being, Self-esteem, Family, Friends, and School. Each item addresses the child’s experience over the past week, and is rated on a 5-point scale (1 = never, 5 = always). Mean item scores are calculated for all subscales and transformed to a 0–100 scale, with 100 indicating very high QoL. Psychometric testing of the KINDL-R revealed good scale utilization and scale fit as well as moderate internal consistency [41]. A Norwegian normative study also confirmed satisfactory internal consistency (alpha = 0.69 to 0.81 for the subscales for 10th graders) and satisfactory test–retest reliability [39]. In our study, there was a large structural missing percentage on the school subscale due to the fact that 29 % of the adolescents in the present study did not attend school. Also, the items of the family subscale, which asked about experiences relating to family life over the past week, did not fit the target group of the present study, and were therefore not applied. Thus, five subscales of the KINDL-R were used in order to measure aspects of QoL.

#### Child Behavior Checklist (CBCL)

The Child Behavior Checklist (CBCL) 6–18 [42] was used for an attrition analysis of the KINDL-R and to assess the relationship between psychopathology and QoL. The CBCL provides comprehensive clinical information concerning emotional and behavioral problems of the child in several domains: total problems, and internalizing and externalizing “syndromes.” The primary contact reported on the adolescent’s emotional and behavioral problems over the preceding 6 months. The CBCL total problems scale consists of 118 items scored on a 0–2 scale; 0 = “Not True”; 1 = “Somewhat or Sometimes True”; 2 = “Very True or Often True,” with a total score range of 0–236. The Norwegian translation of the CBCL has shown satisfactory reliability and validity [43, 44].

### Representativeness of the sample

An attrition analysis was conducted to see if there were any significant differences between participants who had completed the KINDL-R self-report (*n* = 264), and those who had not (*n* = 136) out of the total of 400 participants in the study. This was done by comparing scores on the internalizing and externalizing problem subscale scores of the CBCL. Complete CBCL scores for 240 of the participants with KINDL-R self-reports were available. For those without a completed KINDL-R self-report, the internalizing problem score was available for 114, and the externalizing problem score was available for 116 participants. The results showed no significant difference in the externalizing problem score (mean difference 2.37, *p* = 0.096). There was a significant difference in the internalizing problem score (mean difference 2.35, *p* = 0.036), suggesting that those who did not complete the KINDL-R self-report had a slightly higher internalizing problem score than the rest. However, the mean difference was small compared with the total range on the internalizing problem score of the CBCL (0–64).

### Missing values

Out of the initial 400 participants, 335 completed the CAPA interview, which was used to categorize them according to child maltreatment history. The KINDL-R data files were studied separately for self and proxy reports, and because of the high rate of youth not attending school, this subscale was studied separately from the rest. Participants who did not answer any of the questions were excluded, resulting in the inclusion of 170 respondents for the self-report school subdomain and 264 self-report respondents for the remaining four subdomains. The corresponding proxy reports resulted in 203 respondents for the school subdomain and 303 respondents for the remaining four subdomains. Among these, there were 0.6 to 2.4 % missing values for the self-report school subdomain (four items), 0.4 to 6.1 % for the remaining self-report subdomains (16 items), 0.5 to 2.5 % for the proxy-report school subdomain (four items), and 0.7 to 3.3 % for the remaining proxy-report subdomains (16 items). These missing values were singly imputed, using the expectation-maximization algorithm.

### Statistical analysis

KINDL-R subscale scores for the three groups of adolescents were compared using a general linear model (ANCOVA). Pairwise comparisons were carried out, combining the global F-test with a local least significance difference test to preserve the familywise error rate (FWER) [45]. To compare the KINDL-R subscale scores with the self-reported number of types of adversities, we used linear regression with the latter as an independent variable. Linear regression was also used to study the effects of categories of childhood adversities on KINDL-R subdomains. Both ANCOVA and linear regression were adjusted for age and sex. Confidence intervals for Pearson’s correlation coefficient were based on the Fisher z-transformation. Correlation coefficients of 0–0.29 were considered small, 0.3–0.59 moderate, and 0.6–1 high. Results were considered statistically significant with a two-sided *p*-value <.05. Analyses were carried out in SPSS (v. 21; IBM SPSS).

### Ethics

The Norwegian Regional Committee for Medical and Health Research Ethics approved the present study and the main research project, Mental Health in Adolescent Residents in the Child Welfare System (number of reference 2013/1128/REC Central). Written informed consent was obtained, and if the participant was under 16 years of age, consent from the guardian was also obtained. To avoid making participants feel pressured to participate, the head of the institution received detailed oral and written information about the research project. A six-page standardized information/invitation letter was distributed to the adolescents, and had previously been approved by the Committee for Medical and Health Research Ethics. This information letter described in detail, using simple language, the kind of information to be assessed. It was emphasized that participation in the project was voluntary, that the adolescent did not need to complete all the questions, and that the participant could retract her or his consent at any time. When the research assistant arrived at the institutions the same information was given once again orally to ensure that the adolescent gave informed, voluntary consent to participate in the study.

## Results

Table 1 shows the general characteristics of the adolescents in RYC units and in the general population. Two hundred and thirty-seven adolescents reported exposure to maltreatment, including witnessing violence. Among the adolescents in RYC, the childhood adversity score varied from 0 to 4 (mean = 1.53, SD = 1.12, median = 1).

Table 2 shows the correlation between each of the five subscales on the KINDL-R self-report and the internalizing and externalizing problem scores on the CBCL reported by the primary contact at the unit. As shown, there is a significant small-to-moderate negative correlation between internalizing problems and all five KINDL-R subscales. For the externalizing problem score on the CBCL, we found a small but significant negative correlation between externalizing problems and Friends, Physical Well-being, and Emotional Well-being, but not between externalizing problems and Self-esteem or School.

**Table 2:** Correlation between KINDL-R subdomains and CBCL internalizing and externalizing problems. Correlation coefficient (r) with 95 % confidence intervals

	1	2	3	4	5	6	7
1. Self-esteem	-						
2. Physical well-being	0.562 (0.473 to 0.639)	-					
3. Emotional well-being	0.652 (0.577 to 0.716)	0.560 (0.471 to 0.638)	-				
4. Friends	0.412 (0.307 to 0.507)	0.298 (0.184 to 0.404)	0.475 (0.376 to 0.563)	-			
5. School	0.496 (0.373 to 0.601)	0.449 (0.320 to 0.561)	0.486 (0.362 to 0.593)	0.360 (0.221 to 0.484)	-		
6. CBCL internalizing problems	−0.268 (−0.382 to −0.146)	−0.429 (−0.527 to −0.320)	−0.347 (−0.454 to −0.231)	−0.291 (−0.403 to −0.171)	−0.223 (−0.367 to −0.068)	-	
7. CBCL externalizing problems	−0.068 (−0.193 to 0.059)	−0.193 (−0.312 to −0.068)	−0.188 (−0.307 to −0.063)	−0.143 (−0.265 to −0.017)	−0.131 (−0.282 to 0.027)	0.378 (0.285 to 0.464)	-

Table 3 shows the associations between single categories of childhood adversities and KINDL-R subdomains. Sexual abuse has the strongest association with all five subdomains, and is statistically significant also when adjusted for the other categories of childhood adversities for all but one subdomain (self-esteem). The association between household dysfunction and the subdomains Physical and Emotional well-being is prominent. However, both sexual abuse and household dysfunction display a borderline statistically significant association with the subdomain Self-esteem.

**Table 3:** Associations between categories of childhood adversities and KINDL-R subdomains

	Witnessing violence		Victim of physical violence, family		Sexual abuse		Household dysfunction	
KINDL-R subdomain	B (95 % CI)	Multiadj. B (95 % CI)	B (95 % CI)	Multiadj. B (95 % CI)	B (95 % CI)	Multiadj. B (95 % CI)	B (95 % CI)	Multiadj. B (95 % CI)
Physical well-being	−3.15 (−9.35 to 3.05)	−0.86 (−7.19 to 5.46)	−3.84 (−9.73 to 2.06)	−2.34 (−8.30 to 3.62)	**−8.43 (−15.25 to −1.60)**	**−7.58 (−14.44 to −0.72)**	**−6.50 (−12.23 to −0.77)**	−5.43 (−11.32 to 0.46)
Emotional well-being	**−7.90 (−14.07 to −1.72)**	−6.21 (−12.53 to 0.12)	−1.34 (−7.29 to 4.61)	0.88 (−5.08 to 6.84)	**−8.07 (−14.94 to −1.20)**	**−6.89 (−13.75 to −0.03)**	**−6.91 (−12.66 to −1.15)**	−5.36 (−11.25 to 0.52)
Self-esteem	−1.72 (−8.57 to 5.14)	0.23 (−6.81 to 7.26)	−1.84 (−8.36 to 4.69)	−0.53 (−7.16 to 6.10)	−7.44 (−15.00 to 0.13)	−6.86 (−14.50 to 0.77)	−6.03 (−12.37 to 0.31)	−5.51 (−12.05 to 1.04)
Friends	−2.91 (−8.80 to 2.98)	−1.34 (−7.37 to 4.69)	−3.54 (−9.14 to 2.06)	−2.53 (−8.21 to 3.15)	**−8.81 (−15.28 to −2.34)**	**−8.26 (−14.80 to −1.72)**	−3.04 (−8.52 to 2.44)	−1.80 (−7.41 to 3.81)
School	−5.55 (−12.82 to 1.72)	−4.03 (−11.44 to 3.39)	−4.91 (−11.62 to 1.81)	−3.73 (−10.47 to 3.01)	**−11.36 (−19.17 to −3.54)**	**−10.92 (−18.87 to −2.97)**	0.50 (−5.97 to 6.97)	2.74 (−3.74 to 9.21)

*Note: B* unstandardized regression coefficient, adjusted for sex and age, *Multiadj.* adjusted for sex, age, and all categories of childhood adversities

Statistically significant associations are marked by bold numbers

**Fig. 2:**
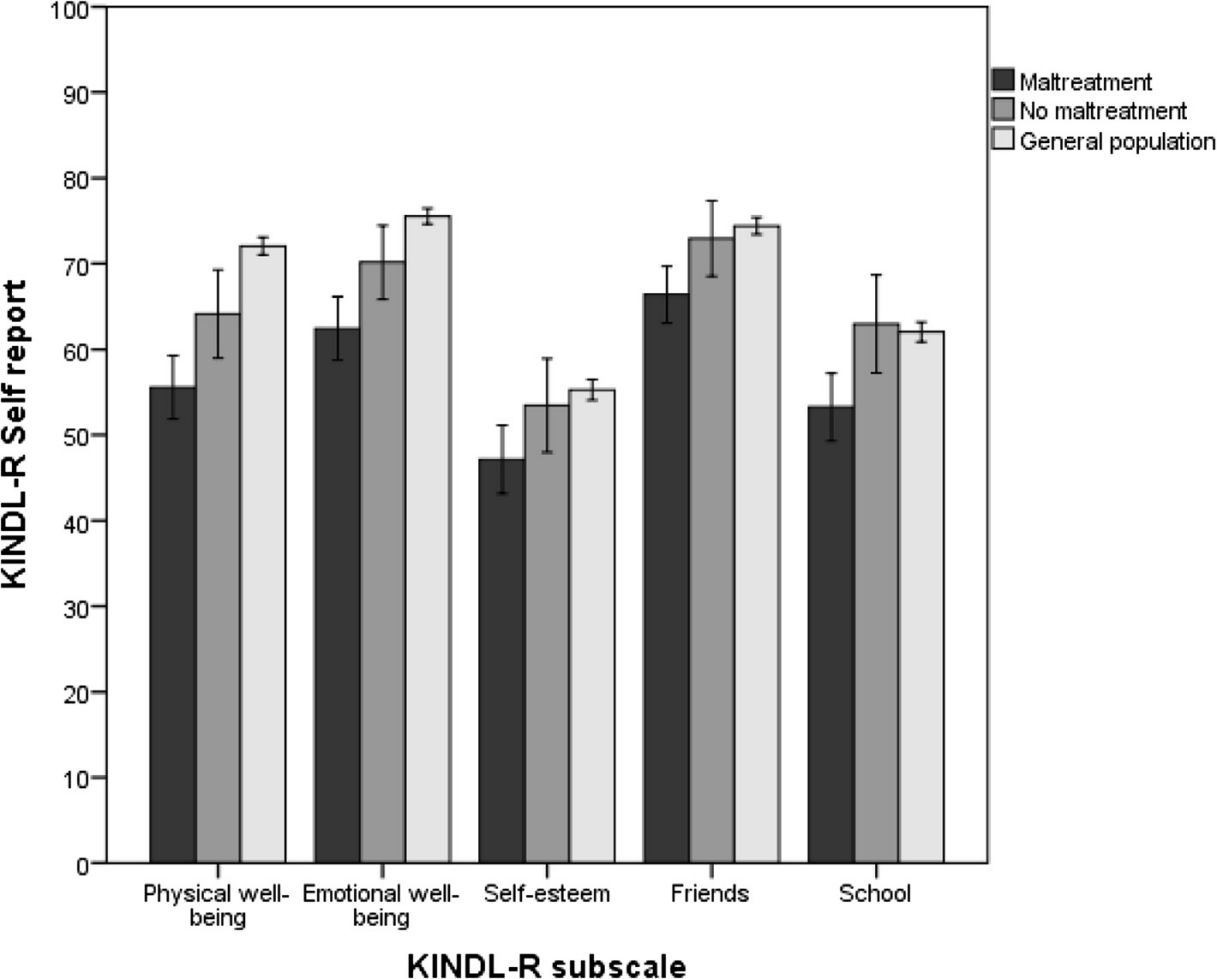
Self-reported quality of life. Estimated mean scores on KINDL-R subdomains (0–100) compared between adolescents in RYC with reported childhood maltreatment, nonmaltreated adolescents in RYC, and adolescents from the general population. Error bars illustrate the 95 % confidence intervals of the mean scores. The subscale scores were compared by using ANCOVA, adjusted for age and sex. Pairwise comparisons were carried out by combining the global F-test with the local least significance difference test to preserve the familywise error rate (FWER). Mean scores, confidence intervals and p-values are presented in table 4 (Appendix 1)

**Fig. 3:**
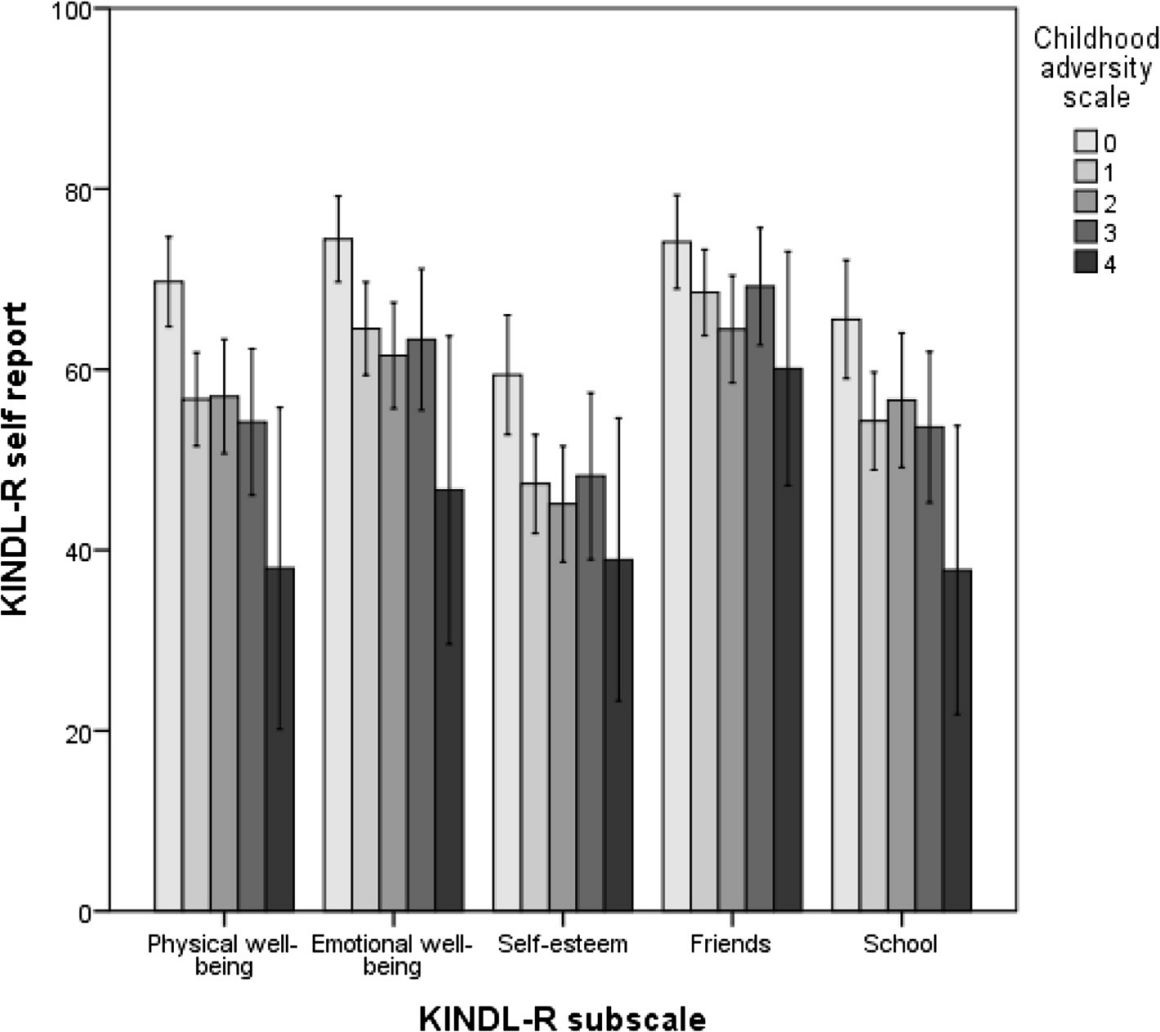
Self-reported quality of life according to the number of types of childhood adversities. The childhood adversity scale is a 4-point scale consisting of the following items: household dysfunction (parental psychopathology, criminality, or alcohol or substance abuse), witnessing violence, victim of family violence, and victim of sexual abuse. Estimated mean scores on KINDL-R subdomains (0–100) were compared between adolescents in RYC with different adversity loads by using linear regression with the childhood adversity scale as the independent variable. The analysis was adjusted for age and sex. Mean scores, confidence intervals and p-values are presented in table 5 (Appendix 2)

QoL self-reports represent the gold standard for measuring adolescents’ QoL. As shown in Fig. 2, adolescents in the maltreatment group evaluated their QoL to be lower on all five subdomains of the KINDL-R. The only results of the overall analysis that were not statistically significant were for the School subdomain. Except for the Self-esteem subdomain, the maltreatment group scores were significantly lower than the reference group in RYC units on all subscales (*p* <0.05). Compared with nonexposed peers in RYC, the 95 % confidence intervals for mean score differences on KINDL-R subdomains (0–100 scale) were 1.9–11.4 (Physical Well-being), 2.2–11.1 (Emotional Well-being), −0.7–10.0 (Self-esteem), and 1.8–10.9 (Friends). Compared with the general population sample, the 95 % confidence intervals for mean score differences were 9.7–17.6 (Physical Well-being), 7.9–15.3 (Emotional Well-being), 3.6–12.5 (Self-esteem), and 5.3–12.8 (Friends). The effects of number of types of adversities are illustrated in Fig. 3. There was a significant decrease in QoL scores for all five subdomains as the number of types of childhood adversities increased.

**Fig. 4:**
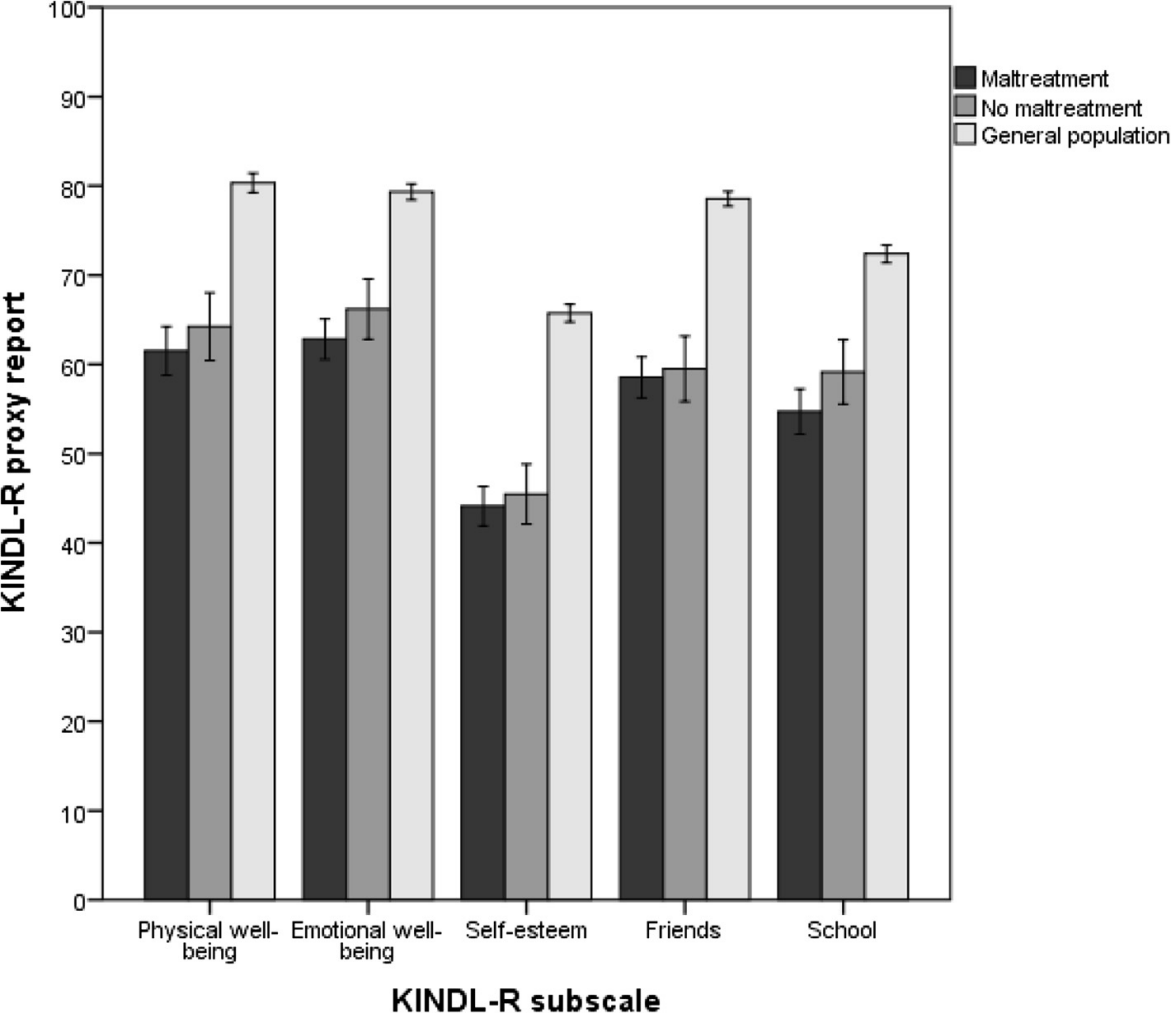
Proxy-reported quality of life. Estimated mean scores on KINDL-R subdomains (0–100) compared between adolescents in RYC with childhood maltreatment histories, nonmaltreated adolescents in RYC, and adolescents from the general population. The reports were completed by adolescents’ primary contacts at the institution (for adolescents in RYC) or parents (for the general population). Error bars illustrate the 95 % confidence intervals of the mean scores. The subscale scores were compared by using ANCOVA, adjusted for age and sex. Pairwise comparisons were carried out by combining the global F-test with the local least significance difference test to preserve the familywise error rate (FWER). Mean scores, confidence intervals and p-values are presented in table 4 (Appendix 1)

**Fig. 5:**
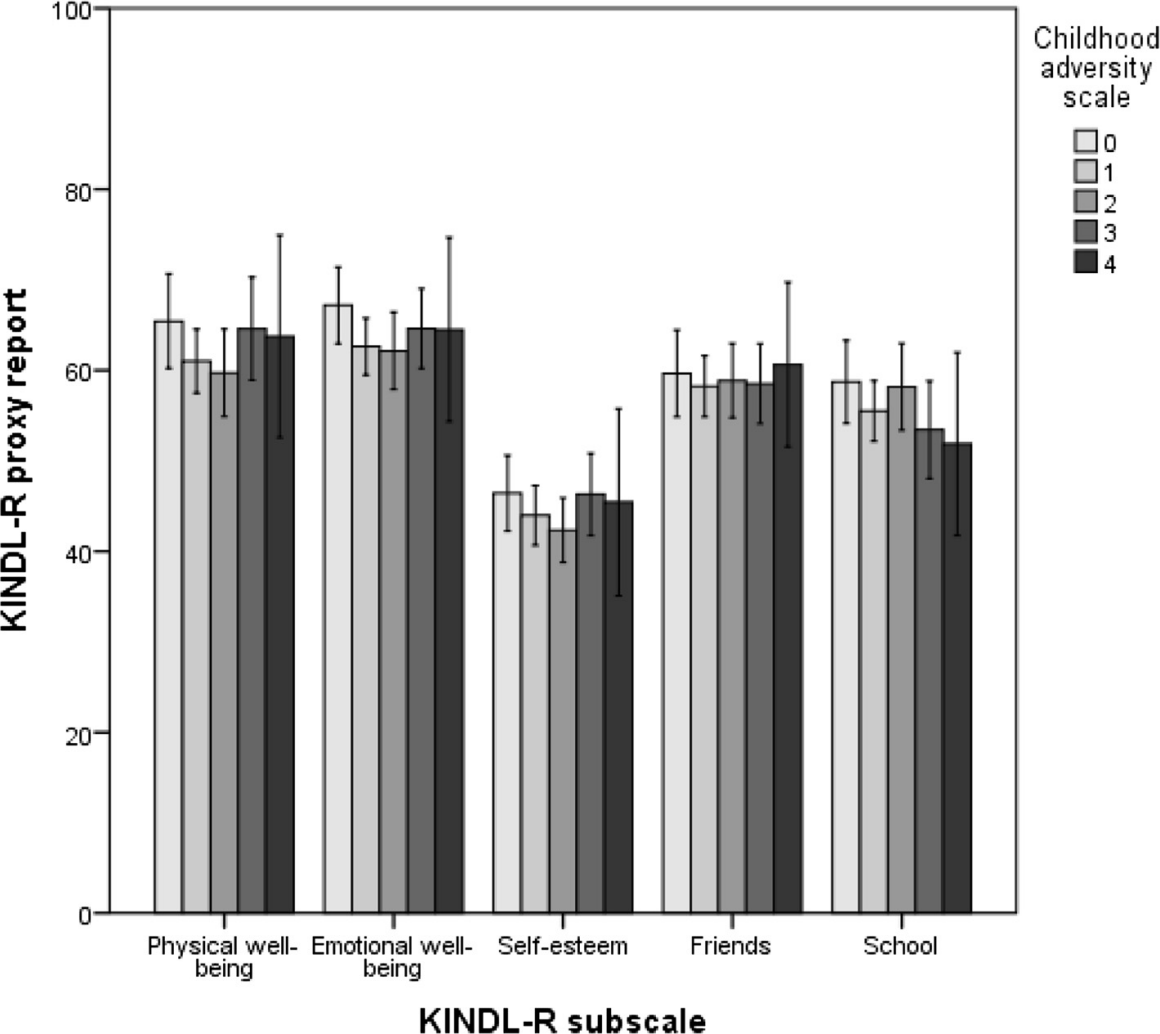
Proxy-reported quality of life according to the number of types of childhood adversities. The childhood adversity scale is a 4-point scale consisting of the following items: household dysfunction (parental psychopathology, criminality, or alcohol or substance abuse), witnessing violence, victim of family violence, and victim of sexual abuse. Estimated mean scores on KINDL-R subdomains (0–100) were compared between adolescents in RYC with different adversity loads by using linear regression with the childhood adversity scale as the independent variable. The analysis was adjusted for age and sex. Reports were completed by adolescents’ primary contacts at the institution (for adolescents in RYC) or parents (for the general population). Mean scores, confidence intervals and p-values are presented in table 5 (Appendix 2)

The proxy reports were scored by the adolescent’s primary contact at the institution, who was the adult with the closest daily-life relationship to the individual youth. Figure 4 shows a significantly lower score for adolescents in both the maltreated and the nonmaltreated group compared with the general population. There were no significant differences between the two groups of adolescents in RYC, in contrast to the results of the self-reports. The differences between the QoL assessments by the adolescents themselves and by their primary contacts in RYC are even more profound regarding number of types of adversities, as illustrated in Fig. 5. We found no significant differences in the QoL score according to the number of types of childhood adversities on the proxy reports.

## Discussion

Adolescents in RYC with child maltreatment histories reported significantly poorer QoL than peers in RYC without maltreatment histories. We also found a dose–response effect in that increasing numbers of types of childhood adversities were associated with poorer QoL on self-reports. As shown in Table 1, there are some differences between our study population and the reference groups. The adolescents in RYC have a higher mean age than the general population sample, and there are more girls in the maltreated group of adolescents. However, this has been taken into account by adjusting for sex and age in the analyses.

### QoL among adolescents in RYC with maltreatment histories compared with nonmaltreated peers in RYC and the general population

Adolescents with maltreatment histories reported lower scores than the reference groups on all five subdomains. The “School” subdomain did not show a significant trend on the global F-test across the three groups compared, but there was a difference in scores between the two groups of adolescents in RYC, as illustrated in Fig. 2. Previous experience of maltreatment seems to affect all five life domains in this population, which might be a key insight for understanding these adolescents’ problems with subjective well-being in several life domains.

### Physical well-being

Physical well-being is a subjective sensation of how the body works and functions. Feelings of illness, pain, low energy, or fatigue are factors that will influence the individual’s physical well-being. We found that adolescents who had experienced maltreatment had a poorer score on the Physical Well-being subdomain than adolescents in our reference groups. This corresponds well with the study of Afifi et al., who reported that child abuse is an important determinant of both physical and mental health-related QoL in adulthood [36], and also with the findings of Lanier et al., who reported that children receiving child welfare services after reports of child abuse had poorer QoL scores on five subdomains (physical health, psychosocial health, emotional functioning, social functioning, and school functioning) compared with a normative sample [37]. Jud et al. did not find significant differences regarding physical well-being in maltreated versus nonmaltreated school-aged children on self-reports [38]. However, their study and control populations were hospital samples, matched for chronic health conditions. A growing amount of research in recent decades has shown that childhood maltreatment is a major risk factor for a broad specter of physical health problems later in life, such as ischemic heart disease, chronic obstructive pulmonary disease, liver disease, obesity, and autoimmune disease [4, 40, 46, 47]. These are major causes of death in adults, and are expensive both for the individual and for society. Adolescents in RYC who experienced childhood maltreatment report poorer physical well-being than peers without these experiences. It may be questioned whether these adolescents receive the medical attention they require, and whether the decline in physical well-being might be preventable. To improve an individual’s physical well-being, several factors could be considered in addition to increased medical care. Aerobic exercise and general physical activity are known to have positive effects on endurance, symptom relief, and well-being [48], and could be easily implemented in RYC units. Ensuring a well-balanced, nutritious diet could also contribute to improved physical well-being [49].

### Emotional well-being

Emotional well-being is a subjective feeling of the degree of happiness, loneliness, boredom, anxiety, and insecurity. The adolescents who reported exposure to maltreatment reported poorer emotional well-being than their peers in the reference groups. These adolescents also had a significantly higher risk of psychiatric diagnoses [24], and our findings are therefore not surprising. The results confirm previous findings, but extend our understanding because the QoL subscale goes beyond pure psychopathology. Easy access to psychiatric health services for diagnostic assessment and therapy is of major importance for these adolescents, but in addition to this, less comprehensive interventions could also prove helpful within the RYC setting. To improve emotional well-being, physical activity is important [50], but meditative motion therapy, like yoga, tai chi, and qi gong have also shown promising results in some smaller studies [48]. The practice of mindfulness could contribute to improving emotional well-being [51], as could ensuring a healthy diet [49, 52].

### Self-esteem

The exposed group of adolescents reported a poorer level of self-esteem than adolescents from the general population. As shown in Fig. 2, they also reported poorer self-esteem than their nonexposed peers in RYC, but this difference was not statistically significant. Childhood maltreatment reflects the caregiver’s inability to provide the safe and secure environment necessary for healthy development. For many children, this might communicate that they lack value and worth, thereby building a basis for low self-esteem. This could have long-lasting effects. Herrenkohl et al. reported poorer self-esteem among adults for whom child welfare services had reported maltreatment during childhood [53]. Improving self-esteem in adolescents in RYC could therefore make a huge impact. Therapeutic interventions addressing attachment difficulties could be considered, but programs that focus on increasing physical activity and exercise might also contribute to positive development [54, 55].

### Friends

Adolescents in RYC evaluated their relationships with their friends to be poor, compared with the evaluations of adolescents in the general population [26]. In this study, we found that the exposed group of adolescents in RYC rated the quality of their friendships as even poorer than their nonexposed peers in RYC. Establishing relationships with friends is an important developmental task during adolescence. However, the experience of childhood maltreatment might result in negative expectations of all interpersonal relations, impeding the formation of new friendships with peers. In addition, adolescents in RYC have a high mean number of placements outside the family home. This contributes to a large number of broken relationships, making it especially difficult to maintain long-lasting friendships in this population. Minimizing the number of placements of children and adolescents in child welfare is therefore an important goal, and cannot be emphasized enough. Each new placement makes it more difficult to establish long-term relationships and to keep friends over time.

### School

The School subdomain measures how much the individual likes school and finds it interesting, how much the individual worries about grades or the future, and how well the individual feels he or she manages schoolwork. We did not find any overall significant differences between our three comparison groups on this subdomain. However, Fig. 2 illustrates that the exposed group of adolescents report a poorer relationship with school than their nonexposed peers in RYC. This is consistent with the review of Romano et al., where children and adolescents with maltreatment histories were found to exhibit impairments in academic achievement, such as poor performance in a variety of school subjects and according to standardized achievement measures, lower grade point averages, frequent school absences, and higher involvement in special education interventions [9]. Childhood maltreatment can cause disruptions in brain development that cause difficulties with memory, attention, and executive functions, all basic processes necessary for optimizing the ability to learn [56, 57]. Early intervention is essential, because poor school performance can result in long-lasting effects through low expectations of future achievement and success, not only by the individual but also by caregivers and teachers, thereby laying the foundations for future underachievement or failure.

### Number of types of childhood adversities and QoL

Figure 3 illustrates the impact of the number of types of childhood adversities. On all five subdomains, there is a significant negative trend showing that increasing numbers of types of childhood adversities corresponds with a lower self-reported QoL score. This corresponds well with the results of the study by Jernbro et al. [32], who found a linear relationship between the number of types of maltreatment and QoL. Our findings also correspond well with the results of Chan [33], who reported that adolescents who experienced four or more types of victimization had poorer QoL than adolescents exposed to fewer types of victimization.

### Proxy report reliability

The primary contacts of the adolescents in RYC reported significantly lower scores for the adolescents than did the parents of adolescents in the general population on all five subdomains of the KINDL-R, implying that the RYC staff noticed a drop in QoL among adolescents in RYC generally. However, there was no difference between proxy reports of adolescents with and without maltreatment histories in RYC. Furthermore, the proxy reports did not show any significant difference according to the number of types of childhood adversities. This suggests that primary contacts do not notice the negative impact of maltreatment histories and number of types of adversities on different QoL subdomains, which is clear from the self-reports. In a previous study on the same population, significant differences were reported between self- and proxy reports on only the Physical Well-being and Friends subdomains, suggesting that primary contacts could serve as satisfactory substitutes for QoL information when self-reports are unavailable [26]. By contrast, the results of the present study suggest that primary contacts may *not* be reliable reporters of adolescents’ QoL, at least not for adolescents with maltreatment histories.

### Strengths and limitations

This was a large, nationwide study of a high-risk population with a good response rate of 67 %, which made it possible to study associations that would otherwise have been hidden. Because we had CBCL scores for both participants and nonparticipants, we could, in an earlier study of this sample [22], estimate DSM IV diagnoses for 541 adolescents, thereby confirming the representativeness of the 335 youths who actually completed the psychiatric interview. Further, we could compare participants with and without a completed KINDL-R, regarding internalizing and externalizing problems. The results showed no significant difference in externalizing problem scores between participants and nonparticipants; however, those who had completed the KINDL-R had a slightly lower internalizing problem score than those who had not (mean difference 2.35, 95 % CI 0.16–4.55). We also found a significant small-to-moderate negative correlation between internalizing problems and all five KINDL-R subdomains (Table 2). Our results might therefore be slightly underestimated compared with the total sample of adolescents in residential care participating in the study (*n* = 400), because the adolescents’ actual QoL score would be slightly lower than we have reported. We did not have access to information on neglect or emotional or verbal abuse. However, previous research has shown that the prevalence of experienced neglect is increased in populations with other maltreatment histories. Further, in Norway, the official child protection policy is that foster care is the preferred form of placement and RYC is a last resort [58]. Thus, we presume that almost all adolescents in our investigated high-risk population would have experienced at least one form of neglect. This could also contribute to a slight underestimation of our results. Maltreatment histories were based on the adolescents’ self-reports alone, and it is possible they could be biased. We did not use parents as informants, thereby limiting our knowledge of maltreatment histories and also the subdomain “Family.” The cross-sectional design of the study limits our ability to draw conclusions on causality.

## Conclusion

Adolescents in RYC with histories of childhood maltreatment report a poorer QoL than nonexposed peers in RYC and adolescents in the general population on all five QoL subdomains we studied. Exposure to more than one type of childhood adversity is common in this population, and is an additional factor negatively associated with QoL in a dose–response relationship. Maltreatment affects children and adolescents in several life domains, including their mental and physical health, and the short- and long-lasting consequences can be detrimental. The use of QoL measures among this high-risk population could unveil problems in areas that go beyond symptoms and diagnoses, thereby opening up the possibility of offering interventions and preventing a further decline in functioning at an early stage in development. Some interventions could be easily implemented by RYC staff, such as offering increased physical activity or exercise, and improving residents’ diet, while others demand comprehensive cooperation between health services, child welfare services, and schools. We suggest that measures of QoL and maltreatment be included in the evaluation of the health and daily functioning of all adolescents in RYC.

## Appendix 1

**Table 4:** Qol score in exposed and non-exposed adolescents (Figs. 2 and 4)

				Pairwise comparison *p*-value		
	Mean	95 % CI	F-test *p*-value	Maltr	No maltr	Gen pop
Self-reports:						
Physical well-being			<.001			
Maltr	57.96	(54.53, 61.38)		-	.006	<.001
No maltr	64.59	(60.31, 68.87)			-	.003
Gen pop	71.58	(70.34, 72.82)				-
Emotional well-being			<.001			
Maltr	63.71	(60.52, 66.90)		-	.003	<.001
No maltr	70.33	(66.35, 74.32)			-	.025
Gen pop	75.30	(74.15, 76.45)				-
Self-esteem			.002			
Maltr	47.34	(43.48, 51.21)		-	.090	<.001
No maltr	52.00	(47.17, 56.83)			-	.210
Gen pop	55.36	(53.96, 56.75)				-
Friends			<.001			
Maltr	65.58	(62.30, 68.86)		-	.006	<.001
No maltr	71.95	(67.85, 76.05)			-	.240
Gen pop	74.62	(73.43, 75.80)				-
School			.067			
Maltr	59.12	(55.03, 63.20)		-	.021	.358
No maltr	66.26	(61.00, 71.51)			-	.071
Gen pop	61.20	(60.00, 62.40)				-
Proxy-reports:						
Physical well-being			<.001			
Maltr	64.30	(61.29, 67.32)		-	.461	<.001
No maltr	65.91	(62.02, 69.79)			-	<.001
Gen pop	79.46	(78.15, 80.78)				-
Emotional well-being			<.001			
Maltr	62.96	(60.41, 65.51)		-	.072	<.001
No maltr	66.27	(62.99, 69.56)			-	<.001
Gen pop	79.28	(78.17, 80.39)				-
Self-esteem			<.001			
Maltr	43.78	(41.09, 46.46)		-	.393	<.001
No maltr	45.43	(41.97, 48.89)			-	<.001
Gen pop	65.83	(64.66, 67.00)				-
Friends			<.001			
Maltr	56.83	(54.39, 59.27)		-	.422	<.001
No maltr	58.24	(55.09, 61.38)			-	<.001
Gen pop	79.09	(78.03, 80.16)				-
School			<.001			
Maltr	57.21	(54.26, 60.15)		-	.066	<.001
No maltr	61.22	(57.44, 65.00)			-	<.001
Gen pop	71.82	(70.78, 72.86)				-

## Appendix 2

**Table 5:** QoL by childhood adversity score (Figs. 3 and 5)

	Childhood adversity score	Mean	95 % CI	*p*-value
Self-report				
Physical well-being				.004
	0	69.70	(64.75, 74.66)	
	1	56.69	(51.49, 61.89)	
	2	57.01	(50.67, 63.34)	
	3	54.17	(46.05, 62.28)	
	4	37.98	(20.17, 55.79)	
Emotional well-being				.002
	0	74.43	(69.66, 79.20)	
	1	64.52	(59.37, 69.68)	
	2	61.54	(55.67, 67.40)	
	3	63.30	(55.47, 71.14)	
	4	46.63	(29.56, 63.71)	
Self-esteem				.046
	0	59.39	(52.76, 66.02)	
	1	47.32	(41.85, 52.79)	
	2	45.09	(38.68, 51.51)	
	3	48.19	(38.96, 57.41)	
	4	38.94	(23.28, 54.60)	
Friends				.015
	0	74.12	(68.91, 79.32)	
	1	68.51	(63.75, 73.27)	
	2	64.46	(58.52, 70.39)	
	3	69.17	(62.69, 75.66)	
	4	60.10	(47.15, 73.05)	
School				.027
	0	65.54	(59.01, 72.08)	
	1	54.29	(59.37, 69.68)	
	2	56.60	(49.12, 64.08)	
	3	53.61	(45.22, 61.99)	
	4	37.78	(21.80, 53.76)	
Proxy-report				
Physical well-being				.257
	0	65.42	(60.21, 70.63)	
	1	61.02	(57.47, 64.56)	
	2	59.72	(54.86, 64.57)	
	3	64.59	(58.90, 70.28)	
	4	63.74	(52.55, 74.93)	
Emotional well-being				.980
	0	67.17	(62.96, 71.39)	
	1	64.52	(59.48, 65.78)	
	2	62.14	(57.87, 66.41)	
	3	46.28	(60.22, 69.00)	
	4	64.50	(54.32, 74.68)	
Self-esteem				.898
	0	46.42	(42.24, 50.59)	
	1	43.95	(40.66, 47.24)	
	2	42.32	(38.77, 45.87)	
	3	46.28	(41.75, 50.81)	
	4	45.42	(35.08, 55.76)	
Friends				.325
	0	59.66	(54.85, 64.46)	
	1	58.22	(54.83, 61.61)	
	2	58.86	(54.75, 62.97)	
	3	58.52	(54.11, 62.93)	
	4	60.61	(51.51, 69.71)	
School				.418
	0	58.73	(54.14, 63.31)	
	1	55.50	(52.14, 58.85)	
	2	58.17	(53.36, 62.98)	
	3	53.41	(48.00, 58.81)	
	4	51.88	(41.76, 61.99)	
